# Supplementary material for: A UMLS-based spell checker for natural language processing in vaccine safety
Source: BMC Med Inform Decis Mak. 2007 Feb 12;7:3. doi: 10.1186/1472-6947-7-3 (PMC1805499; doi:10.1186/1472-6947-7-3)
Supplement: Additional file 13 — RAP application directory. Sets up directory for RAP (RDF application for PHP) source code [file 1472-6947-7-3-S13.gz › rap/doc/phpdoc/__filesource/fsource_dataset__datasetTriXSerializer.php.html]

xml version="1.0" encoding="iso-8859-1"?


File Source for TriXSerializer.php


# Source for file TriXSerializer.php

Documentation is available at TriXSerializer.php

```
1. <?php
2. // ----------------------------------------------------------------------------------
3. // Class: TriXSerializer
4. // ----------------------------------------------------------------------------------


8. /**
9. * Temporary implementation of a TriX-Serializer
10. *
11. *
12. * <BR><BR>History:<UL>
13. * <LI>06-02-2005                : First version of this class.</LI>
14. *
15. * @version  V0.9
16. * @author Daniel Westphal (http://www.d-westphal.de)
17. *
18. * @package     dataset
19. * @access    public
20. ***/
21. class TriXSerializer
22. {

24. /**
25. * Reference to the graphSet
26. *
27. * @var        object GraphSet
28. * @access    private
29. */
30. var $graphSet;


33. /**
34. * Constructor
35. * Needs a reference to a graphSet
36. *
37. * @param    GraphSet
38. * @access    public
39. */
40. function TriXSerializer(&$graphSet)
41. {
42. $this->graphSet=&$graphSet;
43. }

45. /**
46. * Serialize the dataset to a TriX string
47. *
48. * @return   string
49. * @access    public
50. */
51. function & serializeToString()
52. {
53. return $this->_serialize();
54. }

56. /**
57. * Serialize the dataset to a TriX string and save in file
58. *
59. * @param   string
60. * @access    public
61. */
62. function serializeToFile($fileName)
63. {
64. $serializedString=&$this->_serialize();
65. $handle = fopen($fileName, 'w');
66. fwrite($handle, $serializedString);
67. fclose($handle);
68. }


71. /**
72. * Serialize the dataset to a TriX string
73. *
74. * @return   string
75. * @access    private
76. */
77. function & _serialize()
78. {
79. //Trix header
80. $serializedString=
81. '<?xml version="1.0" encoding="utf-8"?>'.
82. '<TriX xmlns="http://www.w3.org/2004/03/trix/trix-1/">';

84. foreach ($this->graphSet->listGraphNames() as $graphName)
85. {
86. $serializedString.='<graph>';
87. $serializedString.='<uri>'.$graphName.'</uri>';
88. for($iterator = $this->graphSet->findInNamedGraphs(new Resource($graphName),null,null,null); $iterator->valid(); $iterator->next())
89. {
90. $serializedString.='<triple>';

92. $statement=$iterator->current();

94. $serializedString.=$this->_node2string($statement->getSubject());
95. $serializedString.=$this->_node2string($statement->getPredicate());
96. $serializedString.=$this->_node2string($statement->getObject());

98. $serializedString.='</triple>';
99. };
100. $serializedString.='</graph>';
101. };
102. //TriX footer
103. $serializedString.='</TriX>';
104. return $serializedString;
105. }

107. /**
108. * Serialize node to a TriX string
109. *
110. * @param Node
111. * @return   string
112. * @access    private
113. */
114. function _node2string($node)
115. {
116. switch ($node)
117. {
118. case (is_a($node,'BlankNode')):
119. return ('<id>'.$node->getLabel().'</id>');

121. case (is_a($node,'Resource')):
122. return ('<uri>'.$node->getLabel().'</uri>');

124. case (is_a($node,'Literal')):

126. if ($node->dtype!=null)
127. return ('<typedLiteral datatype="'.htmlentities($node->dtype).'">'.$node->getLabel().'</typedLiteral>');

129. if ($node->lang!=null)
130. return ('<plainLiteral xml:lang="'.htmlentities($node->lang).'">'.$node->getLabel().'</plainLiteral>');

132. return ('<plainLiteral>'.htmlentities($node->getLabel()).'</plainLiteral>');
133. }
134. }
135. }
136. ?>
```

Documentation generated on Mon, 4 Jul 2005 17:14:54 +0200 by phpDocumentor 1.3.0RC3
